# Microwave biosensor for the detection of growth inhibition of human liver cancer cells at different concentrations of chemotherapeutic drug

**DOI:** 10.3389/fbioe.2024.1398189

**Published:** 2024-05-13

**Authors:** Jun-Ming Zhao, Yi-Ke Wang, Bo-Wen Shi, Yan-Xiong Wang, Yan-Feng Jiang, Gang-Long Yang, Xiao-Dong Gao, Tian Qiang

**Affiliations:** ^1^ School of Internet of Things Engineering, Institute of Advanced Technology, Jiangnan University, Wuxi, China; ^2^ State Key Laboratory of Biochemical Engineering, Institute of Process Engineering, Chinese Academy of Sciences, Beijing, China; ^3^ Key Laboratory of Biopharmaceutical Preparation and Delivery, Chinese Academy of Sciences, Beijing, China; ^4^ School of Biotechnology, the Key Laboratory of Carbohydrate Chemistry and Biotechnology, Ministry of Education, Jiangnan University, Wuxi, China

**Keywords:** cytotoxicity assay, microwave sensors, live cells, drug concentrations, growth inhibition

## Abstract

Cytotoxicity assays are crucial for assessing the efficacy of drugs in killing cancer cells and determining their potential therapeutic value. Measurement of the effect of drug concentration, which is an influence factor on cytotoxicity, is of great importance. This paper proposes a cytotoxicity assay using microwave sensors in an end-point approach based on the detection of the number of live cells for the first time. In contrast to optical methods like fluorescent labeling, this research uses a resonator-type microwave biosensor to evaluate the effects of drug concentrations on cytotoxicity by monitoring electrical parameter changes due to varying cell densities. Initially, the feasibility of treating cells with ultrapure water for cell counting by a microwave biosensor is confirmed. Subsequently, inhibition curves generated by both the CCK-8 method and the new microwave biosensor for various drug concentrations were compared and found to be congruent. This agreement supports the potential of microwave-based methods to quantify cell growth inhibition by drug concentrations.

## 1 Introduction

Cytotoxicity assays are pivotal in evaluating cellular damage induced by drugs, playing a critical role in the drug development process and safety evaluation (Parboosing et al., 2017; Zhang and Wan, 2022). These assays facilitate the determination of a drug’s safety profile, therapeutic window, and potential side effects, thus informing drug design, judicious usage, and toxicity risk assessment. They are instrumental in detecting adverse effects and providing essential data for the secure administration of drugs (Niles et al., 2008; Vaucher et al., 2010).

The relationship between drug concentration, an independent factor influencing cytotoxicity (Chan et al., 2002; Radko et al., 2013), and cytotoxicity is essential to optimize therapeutic efficacy and minimize adverse effects. Understanding the dose-response relationship is pivotal for researchers to strike a delicate balance between drug efficacy and safety, thereby ensuring judicious drug utilization that curtails potential risks. Cancer cells differ from normal cells in many ways, one of which is that they grow and divide very rapidly. In response to this characteristic, a number of cytotoxic drugs have been developed that target rapidly proliferating cancer cells and inhibit their growth and proliferation by interfering with their DNA synthesis or cell division processes (McQuade et al., 2017; Dongsar et al., 2023). However, cytotoxic drugs do not completely discriminate between cancer cells and normal cells (Tofzikovskaya et al., 2015). Mitomycin-C is an example of a cell cycle-specific chemotherapeutic agent widely used in oncology and cytotoxicity research (Tomasz, 1995; Zhang et al., 2019; Park et al., 2021), predominantly acting on the G2 and M phases to impede DNA synthesis and cell division, thereby arresting cancer cell proliferation. Despite its efficacy against various cancer cell types, mitomycin-c’s potential toxicity to normal cells necessitates rigorous dose regulation and vigilant monitoring for adverse effects. Consequently, assessing the drug concentration-inhibition relationship is an indispensable component of cytotoxicity studies.

To accurately evaluate drug impacts on cell viability, two common techniques are employed: real-time cellular analysis (RTCA) and CCK-8 assays (Cai et al., 2019). RTCA offers real-time, non-invasive monitoring of cellular dynamics, but with limited application scope and higher equipment costs (Yan et al., 2018). Conversely, the CCK-8 assay, a standard in cytotoxicity tests, facilitates straightforward colorimetric measurements and is versatile across various cell lines (Wang et al., 2015; Liu et al., 2018; Wang et al., 2018). However, to ensure a sufficient reaction, the CCK-8 method requires a certain incubation time for the reaction, typically 1∼4 h.

While established cytotoxicity assays like CCK-8 and real-time cellular assays are well-developed, ongoing research is delving into novel assays tailored for diverse cellular contexts and specific experimental requirements. The investigation of cytotoxic mechanisms warrants detailed analysis in certain studies, whereas others prioritize the rapidity and precision of the assay’s readouts. Moreover, optical and electrical measurements can often complement each other’s results in the field of biosensing (He et al., 2023). Microwave biosensors, as a new type of biosensor, are highly sensitive and correspondingly fast (their response time is usually only a few seconds to a few mins), allowing real-time results to be obtained in a short period of time (Narang et al., 2018; Gao et al., 2021). Microwave biosensors’ compactness and lightweight design indeed make them ideal for portable device production. Their seamless integration with electronic circuits, coupled with appropriate algorithms, can lead to intelligent data processing products. To date, no attempt has been made to detect drug cytotoxicity using microwave resonance sensors. If the microwave biosensor can be used for cytotoxicity detection, it can complement the original method in terms of advantages and disadvantages. This wound also further broaden the application areas of microwave detection. Microwave biosensors for measuring cytotoxicity have the advantages of eliminating the need for cell staining, rapid detection, low cost, easy integration with matching circuits, and small sample size. Microwave sensors based on resonant elements are very sensitive to the dielectric constant and loss angle tangent of the surrounding medium (Muñoz-Enano et al., 2020), and have been widely used in the fields of biosensing. Researchers have demonstrated that it has promising applications in bacteria detection (Narang et al., 2018; Jain et al., 2020; Jain et al., 2021), blood glucose detection (Yilmaz et al., 2019; Kandwal et al., 2020; Nazli Kazemi anLight, 2023), and many other areas. Since the key to the endpoint method of evaluating drug cytotoxicity is to determine the number of surviving cells at the end of the experiment (Adan et al., 2016), and there have been studies on the differences in dielectric properties of cell solutions at different concentrations (Chen et al., 2014), it has a certain degree of feasibility to do cytotoxicity testing with microwave sensors.

In this paper, we have designed and fabricated a microwave biosensor based on the integrated passive device (IPD) fabrication technology. IPD integrates different passive components (inductors, capacitors, resistors) in a single subcomponent, which is characterized by a small linewidth, precise substrate control, a high degree of integration and fewer parasitic effects (Yu et al., 2019; Chu et al., 2020). Moreover, IPDs demonstrate enhanced stability compared to capacitive or resistive sensors (Yu et al., 2021). The consolidation of multiple passive components onto a single chip allows IPDs to conserve space, diminish energy consumption, bolster system reliability and accuracy of measurements, and ease the transition to productization. Employing this biosensor, we assessed the impact of concentration on cytotoxicity using HepG2 cells as the model and Mitomycin-c as the chemotherapeutic agent. We determined OD450 values via the CCK-8 assay, which is the biological gold standard (Zhou et al., 2018), as a control group for parallel experiments and verified the feasibility of cytotoxicity experiments using microwave sensors by mapping and comparing the curves of the two groups. In addition, we treated the cells with ultrapure water instead of phosphate buffered saline (PBS) in this experiment to verify the feasibility of this treatment in the microwave biosensor cell number measurement experiments.

## 2 Materials and methods

### 2.1 Sensor design and analysis

**FIGURE 1:**
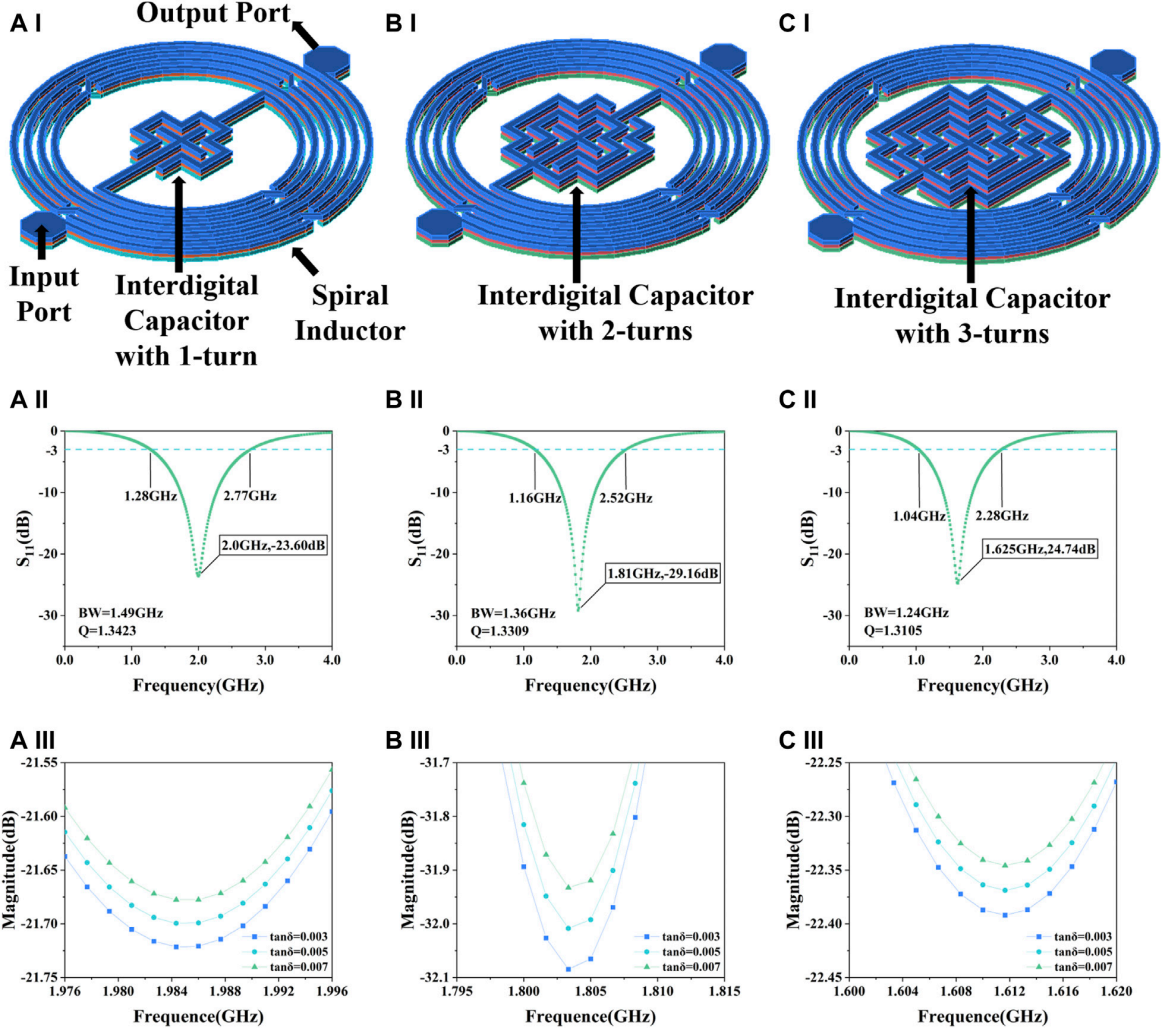
Simulation results of the sensor. **(A)** Interdigital capacitor with 1-turn, **(A–I)** structure, **(A-II)** S_11_, **(A-III)** resonance peak amplitude in different loss angle tangent. **(B)** Interdigital capacitor with 2-turns, **(B–I)** structure, **(B-II)** S_11_, **(B-III)** resonance peak amplitude in different loss angle tangent. **(C)** Interdigital capacitor with 3-turns, **(C–I)** structure, **(C-II)** S_11_, **(C-III)** resonance peak amplitude in different loss angle tangent.

The proposed biosensor is a microwave IPD resonator, consisting of a spiral inductor and an interdigital capacitor, where changes in the electrical parameters of the surrounding medium, mainly the dielectric constant and the loss angle tangent can cause changes in the resonant frequency or the amplitude of the resonance peak. When designing microwave resonators, the relevant parameters and performance are usually adjusted by the capacitance section (Zhu and Abbosh, 2016; Xu and Zhu, 2017). The spiral inductor of the proposed microwave sensor is pre-designed by our group (Wang et al., 2023) and this work focuses on the design, optimization and simulation of the interdigital capacitor. By adjusting the corresponding capacitance structure, we can adjust the frequency sensitivity and amplitude sensitivity of the resonator. In the design of the interdigital capacitive structure, three schemes are considered, respectively, in a cross-shaped central periphery equally spaced increase of 1-turn, 2-turn and 3-turn copper strip lines as shown in Figures 1A–I, B-I, and C-I. The reflection coefficient (S_11_) of the three resonators and the variation of the resonance peak amplitude in different loss angle tangent environments are simulated in the Advanced Design System 2020 (ADS). The Eq. (1) shows that the permittivity of a sample can be obtained by adding the real and imaginary permittivity:

$$\varepsilon_{\gamma }=\varepsilon_{\gamma }^{\prime }+\varepsilon_{\gamma }^{\prime \prime }$$ (1)

The loss angle tangent is calculated from Eq. 2:

$$\tan\delta =\varepsilon_{\gamma }^{\prime \prime }/\varepsilon_{\gamma }^{\prime }$$ (2)

Samples with varying cell concentrations can be characterized by using different values of the loss angle tangent. A change in the loss angle tangent indicates a change in the complex dielectric constant, which in turn affects the S_11_ of the microwave resonator. It can be seen from Figures 1A–II, B-II, and C-II that as the number of turns increases, the resonant frequency decreases, the bandwidth decreases and the Q value decreases. High Q represents high energy storage capacity and frequency selectivity. In terms of sensitivity to the loss angle tangent, the structure of 2-turn shows the best performance as illustrated in Figures 1A–III, B-III, and C-III. Since the resonance amplitude is usually the preferred metric for this type of detection relative to the resonance frequency (Jain et al., 2021), and combined with factors such as the size of the detection area, interdigital capacitor with 2-turns was finally selected as the biosensor.

**FIGURE 2:**
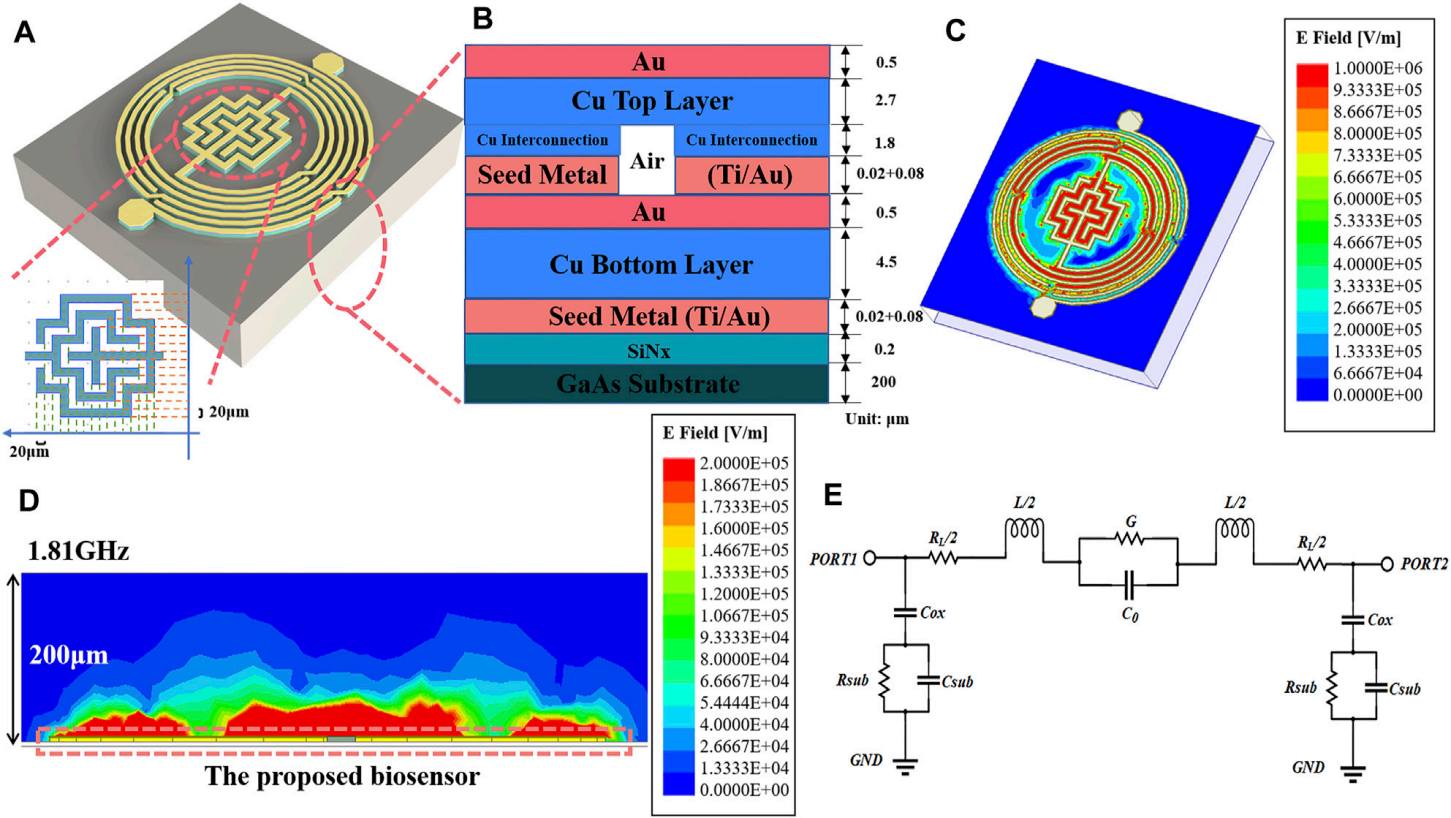
Device structure analysis and electric field simulation. **(A)** Overall device structure and dimensions of interdigital capacitance. **(B)** The hierarchical structure of the device. **(C)** Surface electric field distribution of devices. **(D)** Vertical electric field distribution. **(E)** Equivalent circuit diagram.

Figure 2A delineates the capacitive section’s architecture and precise dimensions. Encircling the device, a spiral inductor integrated with air-bridge structures is observed, while at its nucleus lies an interdigital capacitor, composed of strip wires coiled around a cruciform framework. The strip lines boast a uniform size and interspace of 20 μm. Figure 2B shows the longitudinal layer structure of the sensor, from top to bottom, with a 4.5/0.5 µm Cu/Au top layer, a 1.8 μm copper interconnect layer containing air bridge structure which were introduced in the spiral inductor to increase the mutual inductance and decrease the signal transmission loss in the inductor, a 4.5/0.5 µm Cu/Au bottom layer, a 0.2 μm thick nitride dielectric layer with relative dielectric constant of 7.5 and a loss angle tangent of 0.0036, a 200 μm thick GaAs substrate layer with relative dielectric constant of 12.85 and a loss angle tangent of 0.0028. For the fabrication of our proposed biosensor, seed metal (Ti/Au) is sputtered with the thicknesses of 20/80 nm as for strengthened metallic adhesion. In the electroplating process, gold and copper are tightly bonded through the plating process and have excellent corrosion resistance and do not easily diffuse into the solution, thus they do not interfere with the cytotoxicity analysis of cells. The electric field condition of this resonator is simulated in High Frequency Structure Simulator 19.1(HFSS), and its horizontal E-field strength is shown in Figure 2C, where the E-field strength reaches 10^6^ V/m in its core sensitive region. The highest electric field strengths reported so far in the paper are around 10^5^ V/m (Zarifi et al., 2017; Kumar et al., 2020). The device’s notably higher electric field strengths suggest enhanced penetration and sensitivity. Considering the actual measured solution droplet size, the longitudinal field strength distribution is also simulated, and the results are shown in Figure 2D, illustrating that the sensitive region can still achieve an electric field strength of 10^5^ V/m at a height of 50 μm. High electric field strength in the horizontal and vertical directions reveals the good penetration capability of the device. Consequently, this allows for the use of larger droplet volumes when applying sample droplets, effectively minimizing random sampling errors. Figure 2E shows the equivalent circuit diagram of the device. The capacitance of the oxide layer between the base and the metal can be denoted as *C*_*ox*_, the resistance between the substrate and the ground can be denoted as *R*_*sub*_, the capacitance can be denoted as *C*_*sub*_, the parasitic resistance of the inductor can be denoted as *R*_*L*_, the parasitic conductance of the capacitance can be denoted as *G*. Through the equivalent circuit transformation, the whole device can be regarded as an LC resonator. The complex dielectric constant properties of the cell solution can be modeled using the Debye equation. The relationship between the measured microwave parameters of the cell solution and the complex dielectric constant can be expressed by Eq. (3) as (Withayachumnankul et al., 2013)

$$\left[\begin{array}{c} △f_{0}\\ △\left|S_{11}\right| \end{array}\right]=\left[\begin{array}{cc} m_{11} & m_{12}\\ m_{21} & m_{22} \end{array}\right]\left[\begin{array}{c} △\varepsilon^{\prime }\\ △\varepsilon^{\prime \prime } \end{array}\right]$$ (3)

where $△\varepsilon^{\prime }=\varepsilon_{s}^{\prime }-\varepsilon_{r}^{\prime },△\varepsilon^{\prime \prime }=\varepsilon_{s}^{\prime \prime }-\varepsilon_{r}^{\prime \prime },△f_{0}=f_{s}-f_{r}$ and $△\left|S_{11}\right|={\left|S_{11}\right|}_{s}-{\left|S_{11}\right|}_{r}$ are the differences between the sample (with subscript *s*) and the reference (with subscript *r*) values, $m_{11},m_{12},m_{21},m_{22}$ is the parameters to be determined. In this experiment, a change in cell concentration would cause a change in the loss angle tangent, thus causing a change in $△\left|S_{11}\right|$ .

### 2.2 Preparation of biological sample

**FIGURE 3:**
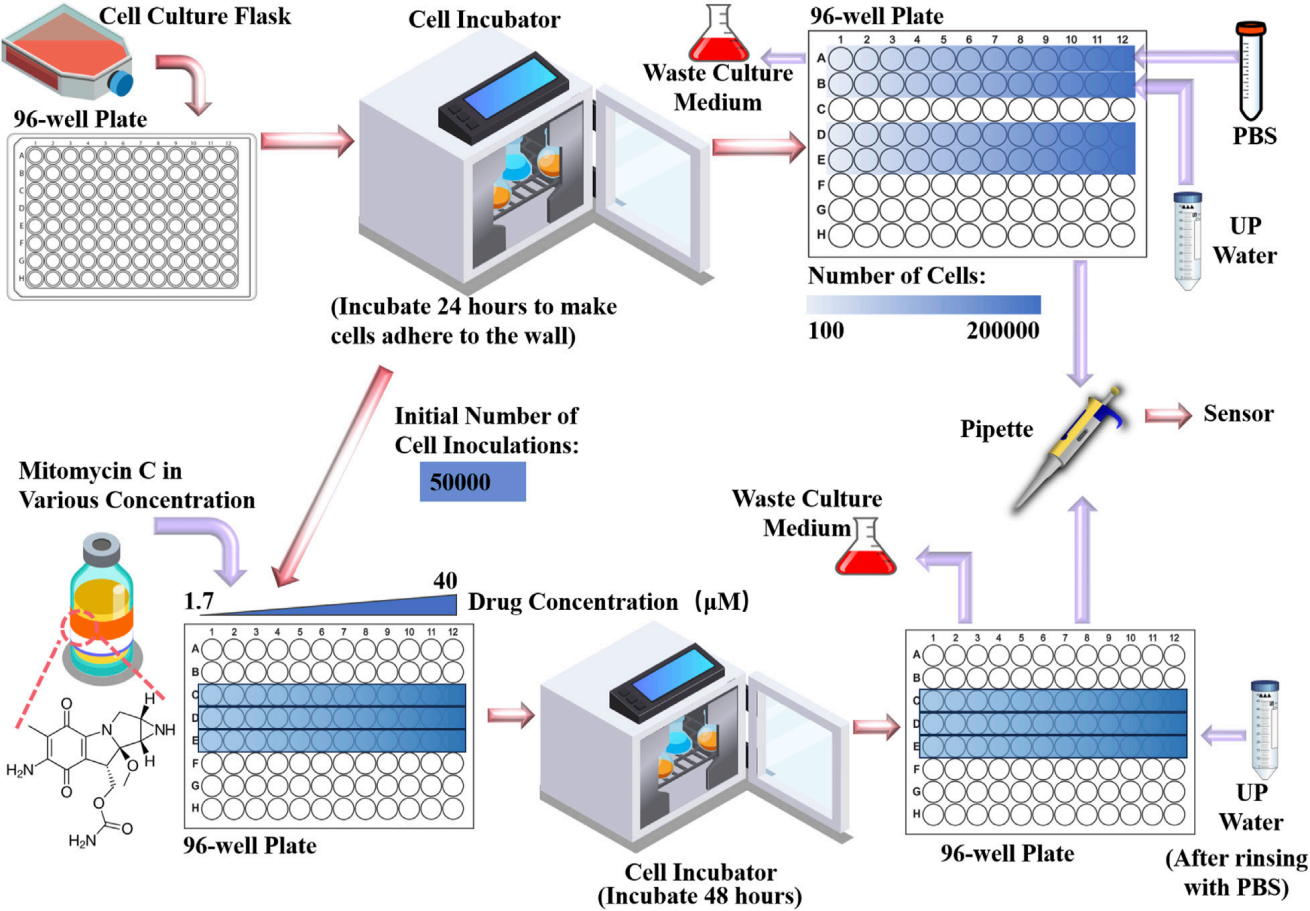
Cell culture and handling, addition of drugs and pre-preparation for measurements.

HepG2 cell line is used as the experimental cells which were purchased from the cell bank of the Chinese Academy of Science (Shanghai, China). It is a human hepatocellular carcinoma cell line commonly used in the study of molecular mechanisms, drug screening and treatment of liver cancer (Elkady et al., 2022). The entire experimental procedure is illustrated in Figure 3. After completing the cell resuscitation, we first performed a pre-experiment using the fabricated microwave biosensor for cell number measurement. We inoculated cells into rows A, B, D, and E of a 96-well plate with a concentration gradient of 100 cells per well to 200,000 cells per well in two-fold increments, and added.

Dulbecco’s modified eagle medium (DMEM) to make them adherent to the bottom by incubating them for 24 h in a CO_2_ incubator at 37 °C. After removing them from the incubator, we pipetted the DMEM from the A and B rows and washed them with PBS. After that, in row A, trypsin treatment was used to dissociate the cells from the bottom, and then 100 μL of PBS was injected into each well; in row B, the same trypsin treatment was used, and then 100 μL of ultrapure water was injected into each well. Rows D and E are used as backup groups. The PBS, trypsin solution and DMEM used in the experiments were purchased from Sangon Biotech (Shanghai, China). The cell culture incubator was purchased from Thermofishe (United States). Normally, in cell number experiments, cells are treated in PBS.In this study, ultrapure water was utilized to treat Group B based on several key considerations. In order to maintain an isotonic state with the cytosol, the ionic concentration of PBS buffer and cytosol is similar. This would result in the cells and the PBS potentially exhibiting similar electrical parameter characteristics, which will lead to a narrowing of the differences in electrical parameters caused by the concentration of the cells. Conversely, the contrast in ionic concentration between ultrapure water and the cell solution is likely to amplify the solution’s electrical parameter changes due to cellular quantity. On the other hand, cell water uptake and cell fluid exudation can result in a more uniform ionic distribution of the solution, thus mitigating random errors linked to small sample sizes. After completing the pre-experimental validation, we seeded 50,000 cells per well on a new 96-well plate, inoculated on row C, D and E as three parallel groups, and after 24 h of CO_2_ thermostatic incubation for cell adhesion, drug administration commenced. In this experiment, we used mitomycin-c as an inhibitor of cell growth. Mitomycin-c was selected as the cell growth inhibitor for this experiment. It was initially dissolved in dimethyl sulfoxide before being prepared into a stock solution at various concentrations. This stock solution was then serially diluted with DMEM to create a two-fold concentration gradient ranging from 1.7 μmol/L to 40 μmol/L. Subsequently, 200 μL of DMEM containing varying concentrations of mitomycin-c was added to each well of the 96-well plate and incubated at 37°C in a CO_2_ incubator for 48 h. Repeat the above steps and prepare the same 3 rows of cells on a new 96-well plate, with one set for OD450 optical measurements and the other set for microwave measurements. For microwave measurement groups, remove the DMEM with a pipette, wash it with PBS, inject 100 μL of ultrapure water into each well. After a period of resting, pipette 1.5 μL of solution and drop it on the sensor for detection. Since the proposed microwave biosensor performs cytotoxicity detection mainly by detecting the concentration of ions contained in the cells, it is unable to distinguish between live and dead cells. Therefore, it is important to ensure that dead cells are cleaned as completely as possible before measurement. Additionally, the ions in the drug can also affect the measurements, so it is important to ensure that the drug is completely purified. The mitomycin-c and dimethyl sulfoxide used were purchased from MedChemExpress (Shanghai, China).

### 2.3 Experimental environment

**FIGURE 4:**
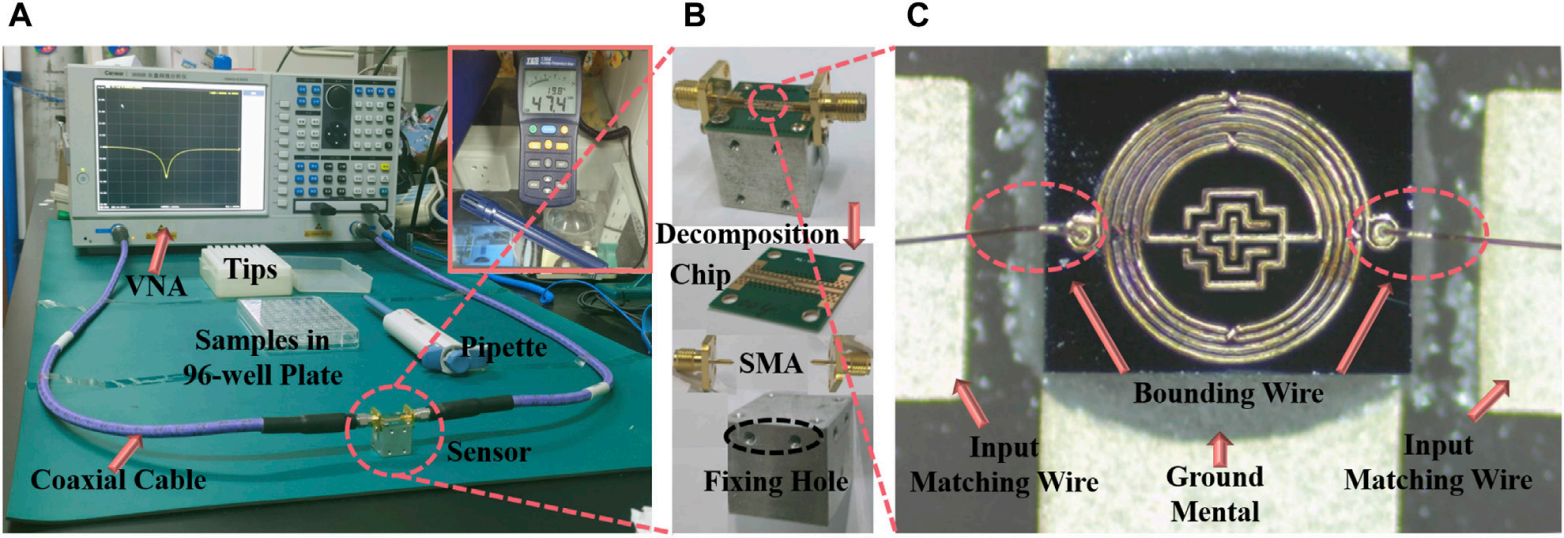
Measuring platforms and fabricated sensor. **(A)** The measurement environment. **(B)** Device structure and assembly schematic. **(C)** Microscope image of the proposed sensor.

The experimental apparatus was positioned on an anti-static mat and comprised a Vector Network Analyzer (VNA, Ceyear, 3656B), the IPD device, coaxial cables, samples, and a pipette, as depicted in Figure 4A. At the heart of the IPD device lies the microwave resonator, detailed microstructurally in Figure 4C. The resonator’s two ports are connected by bonding wire to the corresponding input and output matching wires on the printed circuit board. Figure 4B schematically illustrates the assembled sensor. Its bottom is an aluminum block with screw holes for fixing holes, and the chip is first fixed on top of the aluminum block by screws, and then connected to the coaxial cable of the VNA through the Small A Type connector fixed on both sides. This meticulous assembly ensures the chip remains horizontally stable, mitigating positional errors. The coaxial cable itself is taped to the table to reduce measurement disruptions from any movement. During the measurement, 1.5 μL of solution was added to the middle sensing area with a pipette. To ensure uniform distribution and mitigate the risk of sample settling, each sample drawn from a 96-well plate via pipette is agitated by employing a larger pipette tip. In subsequent sample drops, it was found that when the droplet volume was equal to 2 μL or larger, the droplets were easy to be dispersed irregularly on the surface of the device leading to measurement failure due to destruction of the surface tension of the droplets. After each measurement, the liquid was sucked up with absorbent paper and was cleaned several times with ultrapure water to return the S_11_ to the initial values to ensure that the next experiment was not affected. Since temperature and humidity affect the performance of semiconductor devices, we control and measure the temperature and humidity values, the measurements were carried out at an ambient temperature of 20°C∼21 °C and a humidity of 47 %RH∼48 %RH.

## 3 Results and discussion

### 3.1 Pre-experimental results of cell number measurements

**FIGURE 5:**
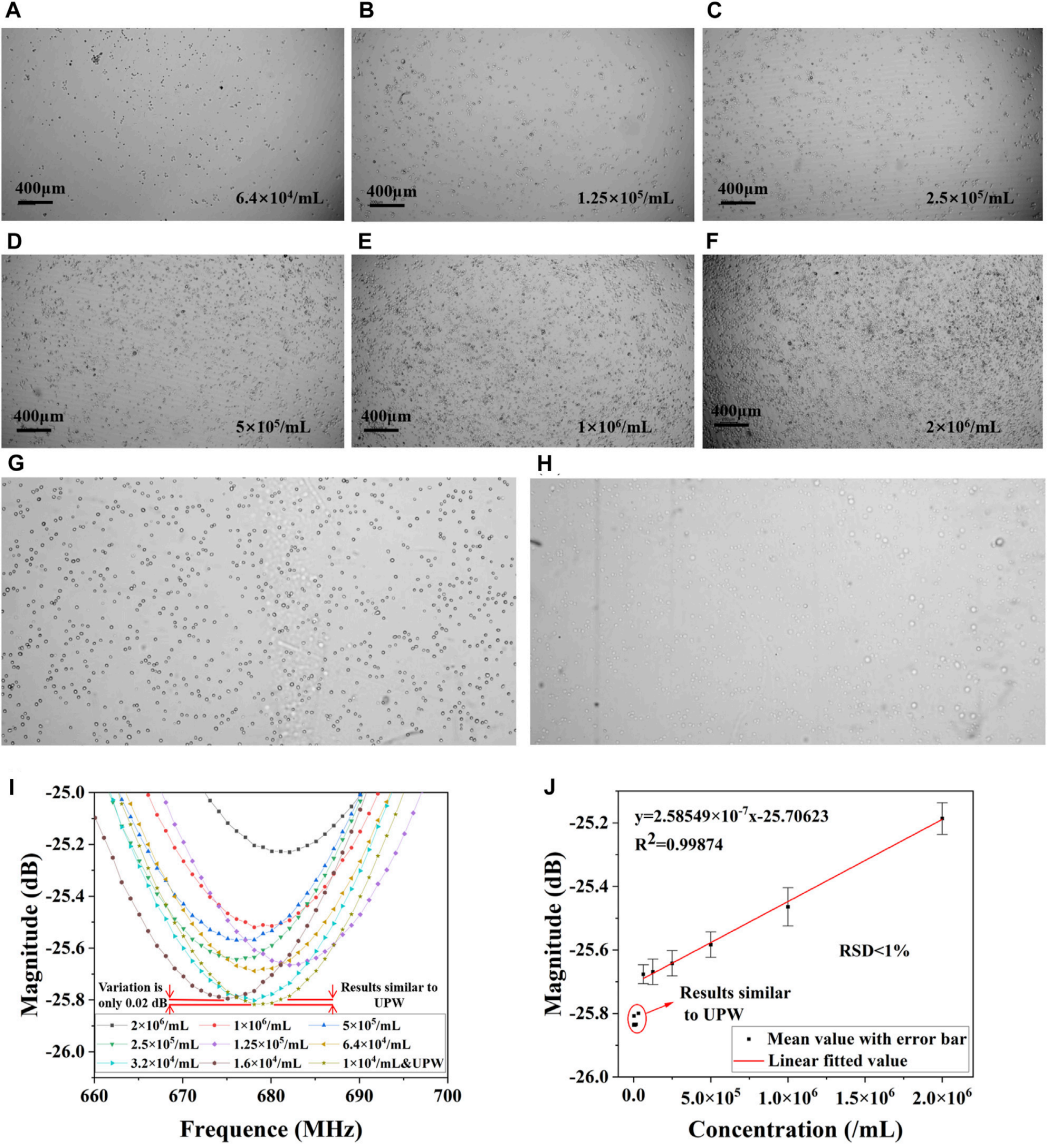
Results of the pre-experiment on cell number measurement. Cells cultured in DMEM at a concentration of **(A)** 6.4×10^4^/mL, **(B)** 1.25×10^4^/mL, **(C)** 2.5×10^5^/mL, **(D)** 5×10^5^/mL, **(E)** 1×10^6^/mL, **(F)** 2×10^6^/mL. **(G)** Cells in PBS, and **(H)** cells in ultrapure water. **(I)** S_11_ of different concentrations in ultrapure water. **(J)** Linear fitted results of cell concentration and magnitude in resonant frequency.

Figure 5 shows the overall results of the cell number measurement experiment. In the cell number measurement pre-experiment, pictures of cells with concentration gradients from 6.4×10^4^/mL to 2×10^6^/mL were taken under the microscope as shown in Figure 5A∼F, which showed healthy growth and a clear concentration gradient. The cells with a concentration gradient from 1×10^4^/mL to 3.2×10^4^/mL did not show a marked difference due to the limited cell numbers, similar to 6.4×10^4^/mL. Figure 5G illustrates the cellular morphology in PBS, where a transition from wall-adherent irregular shapes to more defined round or ovoid forms is observed, predominantly existing as either single entities or aggregated clusters. Under this circumstance, a dynamic equilibrium of ion and water molecule exchange is established between the intracellular and extracellular environments, resulting in comparable ion concentrations. Figure 5H shows the status of the cells after 5 mins of exposure to ultrapure water. Due to the lower osmotic pressure of pure water compared to the cells, water enters the cells, causing them to swell or even dissolve. Cells lose their original morphological characteristics in pure water and become flattened, deformed or ruptured. This can lead to spillage of cell contents dispersed in ultrapure water. The measurements of the S_11_ near the resonance peak of ten quantities of cells after treatment with ultrapure water are shown in Figure 5I. The peak value of the S_11_ decreases with the increase of cell concentration. These measurements were plotted as points in Origin. It can be found that when the number of cells is too low (lower than 6.4 × 10^4^ /mL), the measurements of microwave amplitude are similar, showing a deviation from the other groups and are similar to the measurements of ultrapure water. This may be due to inadequate cytosol exchange with external components when cell numbers are low, and the aspirated 1.5 μL solution may not contain cell membrane components. After selecting the mean of multiple measurements, we performed a linear fit to the mean data on the last six data as shown in Figure 5J. The error bars are based on the mean value, and the relationship between the amplitude of the resonance peak and the concentration of the cells can be characterized by y = 2.58549 × 10^−7^x-25.70623. R^2^ is 0.99874, showing a good linear relationship. The corresponding detection and quantification limits (LOD&LOQ) of the proposed devices was calculated on the basis of following Eqs (4, 5) (Qiang et al., 2017) as 1.41×10^5^/mL and 4.23×10^5^/mL, respectively.

$$\text{LOD}=3.3\times \frac{\text{SD}}{m}$$ (4)

$$\text{LOQ}=10\times \frac{\text{SD}}{m}$$ (5)

where SD is the standard deviation of the frequency response and m is the slope of the regression line. That means, the lowest amount of analyte in a sample which can be detected but not necessarily quantitated as an exact value is 1.41×10^5^/mL, the lowest amount of analyte in a sample which can be quantitatively determined is 4.23×10^5^/mL. This experiment demonstrated that there is a linear relationship between the magnitude of the amplitude under the microwave resonator and the number of cells. Specifically, when the number of cells exceeds a certain threshold (6.4×10^4^/mL), the solution of adherent cells treated with ultrapure water shows this relationship. This experiment confirms that using this microwave sensor to measure cytotoxicity as an endpoint is feasible.

### 3.2 Measurements from drug inhibition experiments

**FIGURE 6:**
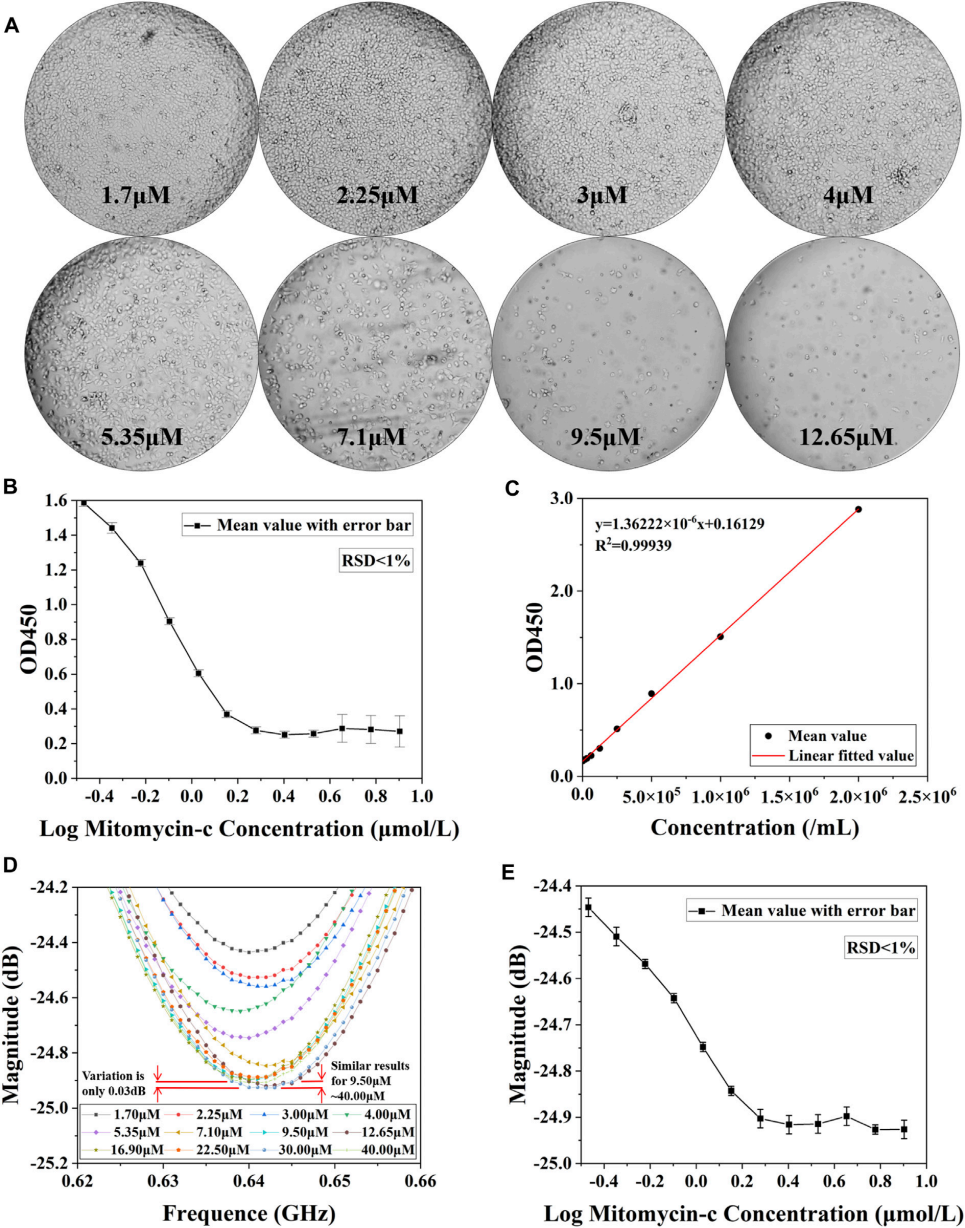
Results of cytotoxicity assay for different concentrations of drugs. **(A)** Microscopic images of cells cultured at different drug concentrations for 48 h and washed with PBS buffer to remove dead cells. **(B)** OD450 value detection of live cells after 48 h of action with different concentrations of Mitomycin-c. **(C)** Measurement results of linear relationship between HepG2 concentration and OD450 value. **(D)** Measurement results of S_11_ after drug concentration 1.7 μM∼40.0 μM action. **(E)** Microwave amplitude detection of living cells in aqueous solutions of Mitomycin-c with different concentrations after 48 h of action.

The results of the drug concentration cytotoxicity assay measurements are presented in Figure 6. Figure 6A demonstrates the cell growth after 48 h of culture with drug concentration ranging from 1.7 μM to 12.65 μM. It can be clearly seen that the number of live cells gradually decreased as the drug concentration increased, and the inhibition of cell growth by the drug can be assessed from the number of surviving live cells. The inhibitory capacity of mitomycin reaches its maximum at drug concentrations of approximately 9.5 μM. Higher concentrations have similar inhibitory effects to 9.5 μM. The cells were subjected to OD450 measurements, depicting the curves in Figure 6B. In addition, the curves of cell concentration and OD450 values were measured for HepG2, and the results are shown in Figure 6C which is similar to the results of the OD450 measurement of cell number in Figure 5J. Since OD450 values have a good linear relationship with cell concentration, OD450 measurements can be equated to cell concentration. Microwave resonance peak amplitude measurements were performed after ultrapure water treatment. A set of near-mean measurements was selected and their S_11_ are plotted in Figure 6D. It can be observed that the amplitude of the resonance peak decreases by approximately 0.45 dB as the drug concentration increases from 1.70 μM to 12.65 μM. The relationship between amplitude and drug concentration was plotted in Figure 6E after an equal number of measurements were taken in three parallel groups and the mean value was selected. It can be seen that the microwave resonance amplitude measurements have similar results to the OD450 measurements. At higher drug concentrations, the resonance amplitude tends to a stable value. Therefore, it is feasible to use the resonance amplitude curve as an assessment index of drug toxicity. Various *in vitro* cytotoxicity assays are currently available including chromium release, bioluminescence, impedance, and flow cytometry (Kiesgen et al., 2021), most of which are based on chemical methods such as fluorescent labelling, optical densitometry and radioactivity determination. These methods have their characteristics and scope of application as well as limitations, microwave sensor methods introduce a new possibility for cytotoxicity determination, and their comparison is presented in Table 1. These methods can be divided into two main categories, optical and electrical, covering a wide range of cellular measurements. In terms of device size, microwave biosensors have the advantage of being small. Microwave methods are on a similar scale to flow cytometry in terms of the concentration of cells that can be processed. Microwave methods are characterized by a tiny sample capacity (0.8 μL∼ 2 μL) in addition to inheriting the advantages of electrical methods that do not require staining of cells.

**TABLE 1:** Summaries of existing cytotoxicity assays.

References	Sample	Measurement method	Structure	Number of cells (/mL)	Pre-processing required	Sample volume
Kim, et al. (2020)	K562	Fluorescent marker	Flow cytometry BD FACSCanto II	2×10^5^∼5×10^6^	CFSE-labeled	≥30 μL
Lai, et al. (2017)	A2780, HepG2	Fluorescent probe	Fluorescent microscopy	NA	Incubated with CLFP probe for 30 mins	Need to cover 96-well plate
Koukoulias, et al. (2023)	K562	Chromium release	Gallio flow cytometer	NA	Surface-stained for 15 mins at 4°C	≥10 μL
Kanemaru, et al. (2022)	B16-F1	Impedance method	Real-time cell analyzer	2.5 × 10^3^∼4 × 10^4^	Not Required	NA
Yang, et al. (2016)	NA	Differential pulse voltammetry (DPV)	Electrochemical workstation	NA	Not Required	100 μL
Proposed Biosensor	HepG2	Microwave method	LC Resonator	6.4 × 10^4^∼2 × 10^6^	Not Required	0.8 μL∼2 μL

### 3.3 Experimental principles

**FIGURE 7:**
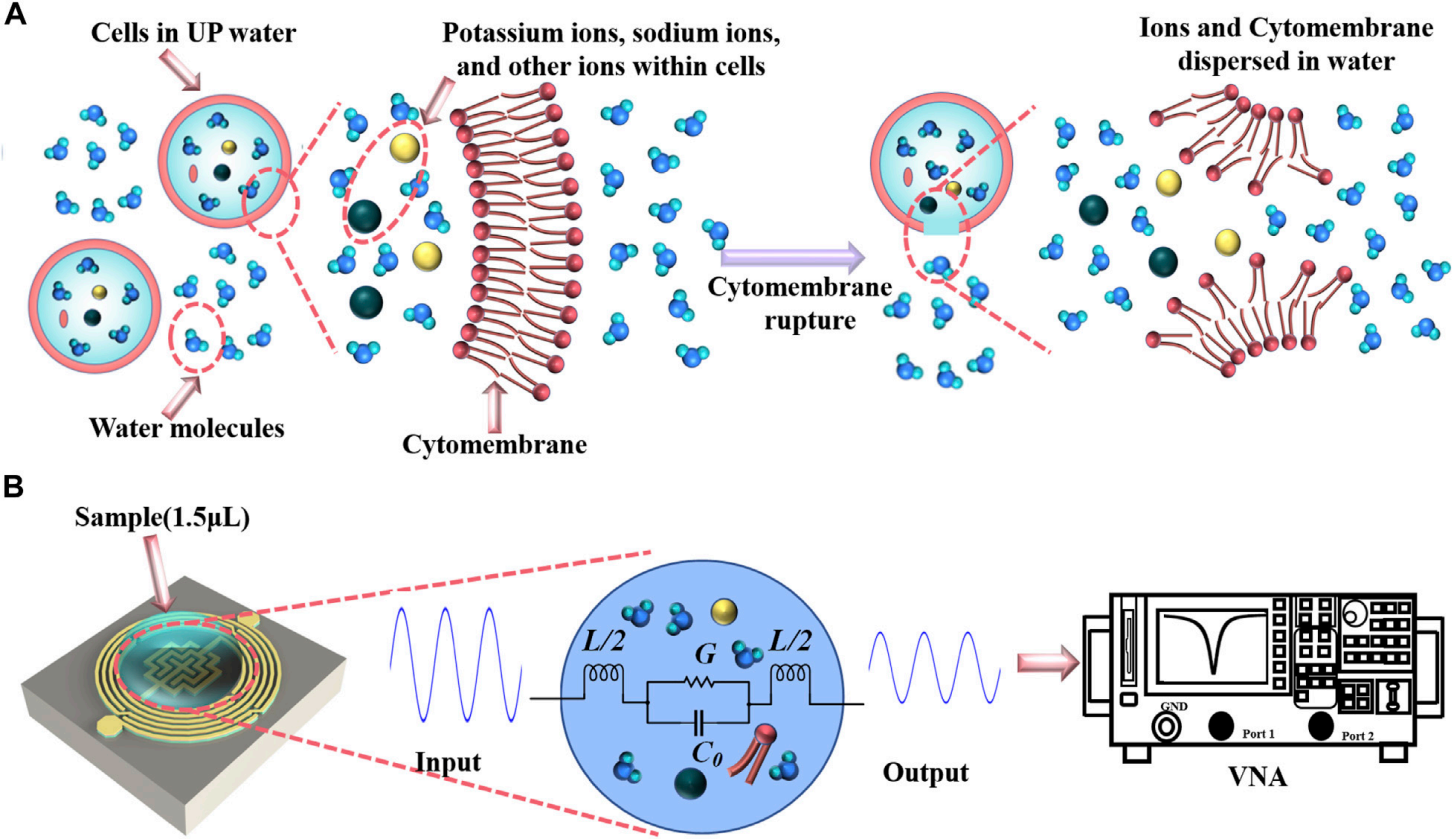
Experimental principles. **(A)** Cell rupture in ultrapure water. **(B)** Measurement of sample in biosensor.

The measurement mechanism of this experiment is divided into two main parts. The first part is cellular water uptake and subsequent rupture as shown in Figure 7A. When a cell is placed in ultrapure water, the concentration of the solution inside the cell is relatively high, while the concentration of the solution in ultrapure water is extremely low. Osmotic forces drive water molecules from the exterior into the cell, causing a volumetric expansion of the cell, a phenomenon termed cellular water absorption. However, if the cell absorbs more water molecules than it can hold, the increased internal pressure may cause the cell membrane to rupture. This typically happens when the cell membrane’s elastic limit is surpassed. After mechanical shaking, the broken cell membrane and various ions within the cell are dispersed relatively uniformly in solution. Differences in the number of cells can lead to differences in the final total ion concentration of the solution, as the cells are treated with equal amounts of ultrapure water. It should be noted that the number of cells should not be too high, otherwise they may not all rupture completely after absorbing water.

The second part is the principle of sample detection by the microwave biosensor as shown in Figure 7B. The cytosol contains a variety of ions, with sodium, potassium and chloride ions making up a large proportion. The effect of ion concentration on dielectric properties has been studied extensively, e.g., an increase in the concentration of sodium chloride leads to a decrease in the loss angle tangent (Wang et al., 2013; Dandan et al., 2015). When the concentrations of sodium chloride and potassium chloride solutions are below a certain value, the dielectric properties of the solutions are similar to those of pure water, and only when they are above a certain value do the dielectric properties show a clear trend (Eldamak et al., 2020). The loss angle tangent describes the nature of the ability of a material to absorb electromagnetic waves and is related to the energy loss in the material. As the concentration of a solution increases, so does the number of solute molecules or ions. At lower concentrations, the ions in the solution have a weaker ability to absorb electromagnetic waves, resulting in a larger loss angle tangent. However, as the concentration increases, the polarization effect of the ions in the solution increases, making the solution less able to absorb electromagnetic waves, resulting in a decrease in the loss angle tangent. Changes in the loss angle tangent affect the degree of microwave attenuation in the solution and the resonance peak of the resonator. When the solution is dropped onto the capacitive area of the microwave resonator, the medium surrounding the capacitive area changes, and the microwave biosensor detects this change sensitively and rapidly. The VNA sends a microwave signal over a set frequency range and measures the amplitude and phase of the reflected and transmitted signals. By varying the frequency and recording the corresponding signal response, data on the S_11_ can be obtained. Further, the VNA can be connected to a computer to efficiently detect changes in the analyzed parameters using the corresponding software.

## 4 Conclusion

In this work, a microwave resonant sensor based on an integrated passive device is presented. The device can be used for cell number detection and further, for the assessment of the degree of cell growth inhibition by drug concentration. The sensor’s capability to detect cytotoxicity was validated against the biological gold standard, the CCK-8 assay. Unlike the usual PBS treatment of cells, ultrapure water was used to treat the cells in this experiment, offering an innovative approach for cell sensing via microwave technology. This novel method provides rapid, precise, and miniaturized cytotoxicity assessments, suitable for various applications. Future enhancements should concentrate on minimizing random detection errors through appropriate peripheral matching circuits and improving sensor sensitivity via structural design modifications. The improvement of the device structure relies mainly on the optimization of the interdigital capacitance. The matching of microwave biosensors with electronic circuits and the introduction of algorithms can result in a miniaturized smart device.

## Data Availability

The original contributions presented in the study are included in the article/Supplementary material, further inquiries can be directed to the corresponding authors.
